# Mail-Order Pharmacy Dispensing of Mifepristone for Medication Abortion After In-Person Screening

**DOI:** 10.1001/jamainternmed.2024.1476

**Published:** 2024-05-13

**Authors:** Daniel Grossman, Sarah Raifman, Natalie Morris, Andrea Arena, Lela Bachrach, Jessica Beaman, M. Antonia Biggs, Amy Collins, Curtiss Hannum, Stephanie Ho, Susan M. Seibold-Simpson, Mindy Sobota, Kristina Tocce, Eleanor B. Schwarz, Marji Gold

**Affiliations:** 1 Advancing New Standards in Reproductive Health, Bixby Center for Global Reproductive Health, Department of Obstetrics, Gynecology & Reproductive Sciences, University of California, San Francisco, Oakland, California; 2 Department of Family Medicine, Brown University, Pawtucket, Rhode Island; 3 Department of Pediatrics, University of California, San Francisco; 4 Division of General Internal Medicine, Zuckerberg San Francisco General Hospital, University of California, San Francisco; 5 Allegheny Reproductive Health Center, Pittsburgh, Philadelphia; 6 Delaware County Women’s Center, Chester, Philadelphia; 7 Highland Hospital, Alameda Health System, Oakland, California; 8 Southern Tier Women’s Health Services, Vestal, New York; 9 Department of Medicine, Alpert Medical School at Brown University, Providence, Rhode Island; 10 Planned Parenthood of the Rocky Mountains, Denver, Colorado; 11 Department of Family and Social Medicine, Albert Einstein College of Medicine/Montefiore Medical Center, Bronx, New York

## Abstract

**Importance:**

Before 2021, the US Food and Drug Administration required mifepristone to be dispensed in person, limiting access to medication abortion.

**Objective:**

To estimate the effectiveness, acceptability, and feasibility of dispensing mifepristone for medication abortion using a mail-order pharmacy.

**Design, Setting, and Participants:**

This prospective cohort study was conducted from January 2020 to May 2022 and included 11 clinics in 7 states (5 abortion clinics and 6 primary care sites, 4 of which were new to abortion provision). Eligible participants were seeking medication abortion at 63 or fewer days’ gestation, spoke English or Spanish, were age 15 years or older, and were willing to take misoprostol buccally. After assessing eligibility for medication abortion through an in-person screening, mifepristone and misoprostol were prescribed using a mail-order pharmacy. Patients had standard follow-up care with the clinic. Clinical information was collected from medical records. Consenting participants completed online surveys about their experiences 3 and 14 days after enrolling. A total of 540 participants were enrolled; 10 withdrew or did not take medication. Data were analyzed from August 2022 to December 2023.

**Intervention:**

Mifepristone, 200 mg, and misoprostol, 800 µg, prescribed to a mail-order pharmacy and mailed to participants instead of dispensed in person.

**Main Outcomes and Measures:**

Proportion of patients with a complete abortion with medications only, reporting satisfaction with the medication abortion, and reporting timely delivery of medications.

**Results:**

Clinical outcome information was obtained and analyzed for 510 abortions (96.2%) among 506 participants (median [IQR] age, 27 [23-31] years; 506 [100%] female; 194 [38.3%] Black, 88 [17.4%] Hispanic, 141 [27.9%] White, and 45 [8.9%] multiracial/other individuals). Of these, 436 participants (85.5%; 95% CI, 82.2%-88.4%) received medications within 3 days. Complete abortion occurred after medication use in 499 cases (97.8%; 95% CI, 96.2%-98.9%). There were 24 adverse events (4.7%) for which care was sought for medication abortion symptoms; 3 patients (0.6%; 95% CI, 0.1%-1.7%) experienced serious adverse events requiring hospitalization (1 with blood transfusion); however, no adverse events were associated with mail-order dispensing. Of 477 participants, 431 (90.4%; 95% CI, 87.3%-92.9%) indicated that they would use mail-order dispensing again for abortion care, and 435 participants (91.2%; 95% CI, 88.3%-93.6%) reported satisfaction with the medication abortion. Findings were similar to those of other published studies of medication abortion with in-person dispensing.

**Conclusions and Relevance:**

The findings of this cohort study indicate that mail-order pharmacy dispensing of mifepristone for medication abortion was effective, acceptable to patients, and feasible, with a low prevalence of serious adverse events. This care model should be expanded to improve access to medication abortion services.

## Key Points

**Question:**

Is medication abortion with mail-order pharmacy dispensing of mifepristone effective, acceptable, and feasible?

**Findings:**

This prospective cohort study included 506 participants and 510 medication abortions (≤63 days’ gestation at enrollment) that were provided through mail-order pharmacy dispensing after an in-person eligibility screening; 97.8% were complete abortions and 91.2% of participants reported satisfaction with medication abortion. Serious adverse events were rare (0.6%) and none were associated with mail-order dispensing.

**Meaning:**

These findings support the US Food and Drug Administration’s decision to remove the in-person dispensing requirement for mifepristone.

## Teaser

This cohort study estimates the effectiveness, acceptability, and feasibility of dispensing mifepristone for medication abortion using a mail-order pharmacy.

## Introduction

Medication abortion with mifepristone and misoprostol is safe, effective, and preferred by many patients.^1^ Between 2020 and 2023, the proportion of nonhospital abortions in the US that were medication abortion increased from 53% to 63%.^2^ This increase likely is related to both the COVID-19 pandemic and the US Supreme Court decision in *Dobbs v Jackson Women’s Health Organization*,^3^ which eliminated federal protections for abortion. In particular, telehealth provision of medication abortion has recently expanded.^4,5,6,7^

Before 2021, the US Food and Drug Administration (FDA) required that, as part of mifepristone’s Risk Evaluation and Mitigation Strategy, it be dispensed in person at a clinic, medical office, or hospital.^8^ During the COVID-19 public health emergency, the FDA suspended the in-person dispensing requirement, allowing clinicians to provide medication abortion by telehealth. In 2021, the FDA reviewed the evidence and recommended permanently removing the in-person dispensing requirement (noting that it was not necessary to ensure safe and effective use of mifepristone), and explicitly allowed for certified pharmacies to dispense the medication. In 2023, the FDA clarified the components of pharmacy certification.^8^

The in-person dispensing requirement for mifepristone had limited the pool of qualified clinicians able to provide medication abortion. A 2016 to 2017 national survey of obstetrician-gynecologists estimated that 14.4% of those who saw patients seeking abortion care had provided medication abortion during the previous year.^9,10^ The study estimated that the proportion of clinicians providing medication abortion would double if they were permitted to prescribe mifepristone through a pharmacy rather than dispense it in-person at a clinic. An important challenge to in-person dispensing for clinicians is the logistics of stocking the medication in their facilities.^10^

The in-person dispensing requirement may be an even greater obstacle for primary care clinicians, including internal medicine and family medicine physicians, who might see only a small number of patients seeking abortion services.^11^ Clinicians in primary care settings have faced opposition from colleagues and administrators when seeking institutional support and approval to stock mifepristone onsite.^11,12^ Yet, many patients say they would prefer to see their primary care practitioner for an abortion.^13,14^ A mail-order dispensing model—in which patients have an in-person visit with the clinician and receive the medications by mail—has the potential to reduce barriers faced by clinicians and achieve patient preferences. However, research on this model has been limited.

In this prospective cohort study, we aimed to estimate the effectiveness, acceptability, and feasibility of providing medication abortion with medications dispensed by a mail-order pharmacy after an in-person eligibility assessment. We compared our findings with published data on medication abortion provided by in-person dispensing of medications. We previously published an interim analysis of the study.^15^

## Methods

This study was conducted under an FDA Investigational New Drug application and was registered with ClinicalTrials.gov.^16^ The institutional review boards of the University of California San Francisco, Albert Einstein College of Medicine (New York, New York), Christiana Care (Newark, Deleware), Kent Hospital (Warwick, Rhode Island), Lifespan (Providence, Rhode Island), and Alameda Health System (Oakland, California) approved the study. Site investigators completed the mifepristone prescriber agreement form, and patients completed the patient agreement form.^8^ Trained staff obtained written informed consent from interested and eligible patients. The design of this study followed the Strengthening the Reporting of Observational Studies in Epidemiology (STROBE) reporting guidelines.^17^

### Study Design and Participants

From January 2020 through May 2022, with a pause from March to June 2020 due to the COVID-19 pandemic, we enrolled patients in a prospective cohort study at 11 clinical sites in 7 US states (California, Colorado, Delaware, Georgia, New York, Pennsylvania, and Rhode Island). The sites included 5 abortion clinics and 6 primary care clinics, 4 of which had not provided abortion care before the study. The study was advertised at meetings and on listservs that included primary care and abortion practitioners across the country. Interested sites were informed that the study would provide training in medication abortion (if applicable) and support integrating the service into their practice. Sites with sufficiently large eligible patient populations and administrative support were selected to participate.

Services were provided by physicians and advanced practice clinicians specializing in family medicine, internal medicine, obstetrics and gynecology, or pediatrics. Participants were eligible for the study if they were seeking and eligible for medication abortion according to the FDA-approved mifepristone label^18^; willing to receive medications from a mail-order pharmacy; willing to use misoprostol buccally as described in the labeling; able to read and speak English or Spanish; willing to be contacted by email or phone; and pregnant with a gestational duration of 63 or fewer days (to reduce the possibility that shipping delays would be associated with mifepristone being used after the FDA limit of 70 days’ gestation). Depending on state parental consent requirements, participants were eligible if they were at least 15 (8 sites), 16 (1 site), or 18 years of age (2 sites).

Study design, recruitment, and procedures were published previously.^15^ Clinicians evaluated patients in person for medication abortion eligibility and determined gestational duration according to the patients’ self-reported history confirmed by physical examination or ultrasonography imaging, consistent with standard protocols.^19,20^ Consenting participants received instructions on medication use and were scheduled for follow-up to confirm abortion completion. Follow-up options included ultrasonography, serum human chorionic gonadotropin measurement, or a telephone call to assess symptoms followed by home urine pregnancy testing. Participants received gift cards at enrollment ($15) and after completing each of 2 surveys ($25). After consenting, participants were informed that the study would pay for the cost of clinical visits, medications, pharmacy dispensing fees, and shipping.

Clinic staff assessed 1339 patients for eligibility and enrolled 540 participants (Figure). Two participants withdrew from the study and obtained medications in clinic, and 538 participants received medications via mail-order pharmacy dispensing. Eight participants did not take the medications they received in the mail. Of the remaining 530 participants, 20 (3.8%) did not follow up with the clinic nor complete survey questions related to abortion completion; they were excluded from the analysis. Four patients participated in the study twice for 2 abortions and were counted twice in the study flow diagram because the unit of analysis was abortions (Figure).

**Figure.**
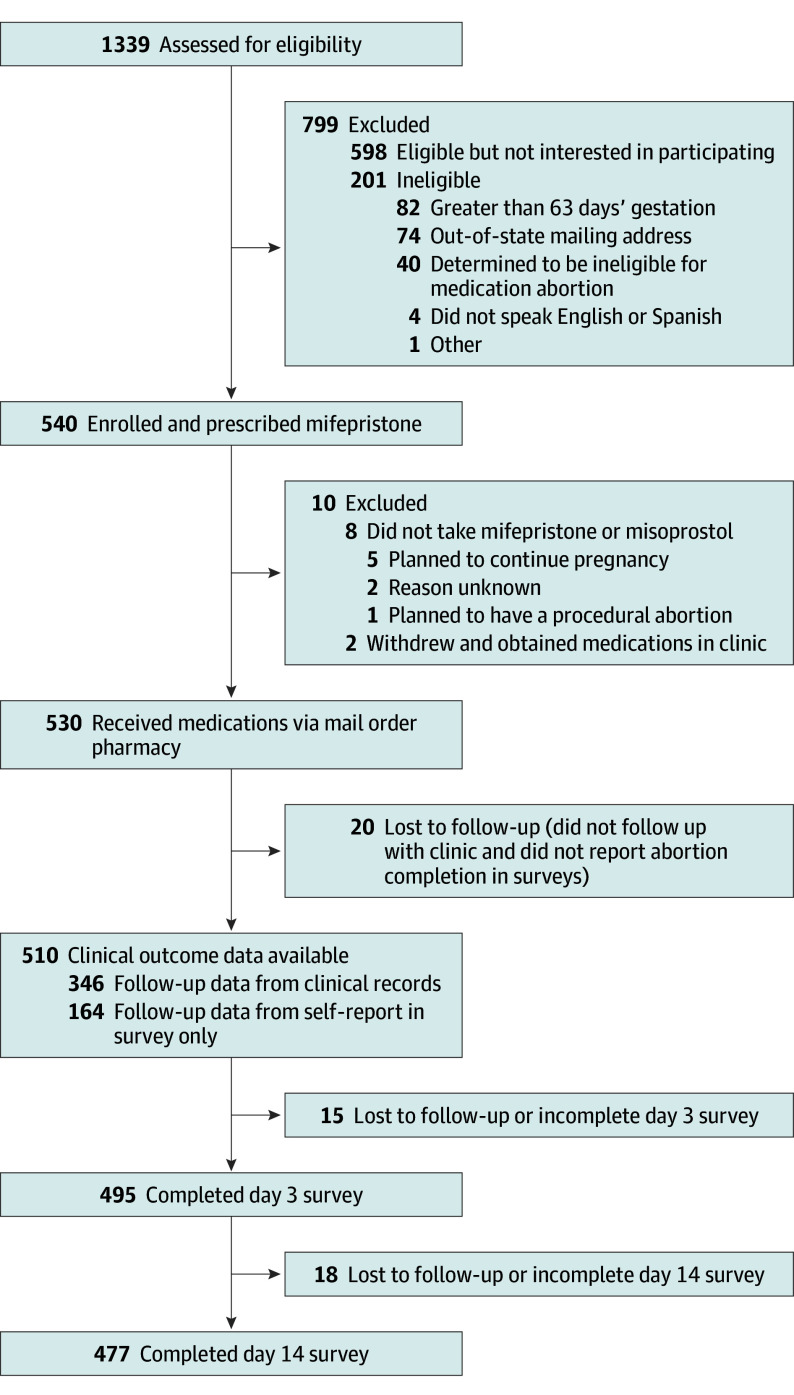
Study Flow of Patients Seeking Medication Abortion Who Were Evaluated in Person and Received Mifepristone From a Mail-Order Pharmacy, 2020 to 2022 The unit of analysis was an abortion (4 patients were enrolled in the study twice).

On enrollment day, clinicians prescribed mifepristone, 200 mg, and misoprostol, 800 µg, to a mail-order pharmacy. At the clinician’s discretion, some also prescribed analgesics, antibiotics, antiemetics, pregnancy tests, and contraceptives. If it was anticipated that the patient would take mifepristone at 63 days’ gestation or later, some clinicians prescribed a second dose of misoprostol, 800 µg, to take 4 hours after the first dose. The mail-order pharmacy processed prescriptions according to a “next-day delivery” workflow and shipped packages (unmarked other than the pharmacy return address) to participants.

On days 3 and 14 after enrollment, we sent participants email requests to complete online surveys (Qualtrics LLC) regarding their experience receiving the package, taking the medications, and having the abortion. Participants provided demographic information, including self-reported race and ethnicity, at the end of the first survey. Six or more weeks after enrollment, personnel from each site entered deidentified data on visits and any other contact with participants into electronic forms (REDCap, Vanderbilt University). Details regarding information collected in each survey were published previously.^15^ We used survey and medical record data to identify abortion outcomes and adverse events (AEs) occurring within 6 weeks of enrollment, including any unscheduled visits for symptoms possibly related to the medication abortion. Serious AEs were defined as death, hospitalization, blood transfusion, or surgery.^21^

### Study Outcomes

Our primary outcomes included effectiveness and acceptability. We evaluated medication abortion effectiveness as the proportion of abortions that were complete (with medications only) within the 6-week study follow-up period. Patient acceptability was measured by the proportion of patients who reported that they were satisfied or very satisfied with the medication abortion, and by the proportion reporting they would use the mail-order service again if they needed another abortion. We also measured the proportion who reported they were very satisfied with the mail-order model. Secondary outcomes included feasibility of the model, determined by the proportion of patients who reported timely (within 3 days following enrollment visit) and confidential delivery of medications, as well as safety outcomes (ie, serious AEs).

### Statistical Analysis

Given low loss to follow-up, we excluded participants with missing outcome data. We calculated binomial 95% CIs for the main outcomes. To assess how the effectiveness and acceptability of the mail-order model compared with that of in-person dispensing, we calculated risk differences with corresponding 95% CIs and *P* values using a statistical test on the equality of proportions for effectiveness and acceptability comparing our sample estimates with published estimates of patients obtaining mifepristone in person. A review of US medication abortion trials with in-person dispensing (N = 16 794)^18^ included in the mifepristone label found a pooled estimate of effectiveness of 97.4%. A meta-analysis of 8 studies found that an average of 88.4% of those who had a medication abortion and took misoprostol at home (N = 3138) reported being “satisfied or highly satisfied.”^22^ A study of medication abortion up to 63 days’ gestation (N = 1080) found that 89.7% of patients would use medication abortion again.^23^ If the lower bounds of the 95% CIs of the risk differences in our estimates were no lower than −0.05 (or 5% worse), we concluded that the mail-order dispensing model was comparable with in-person dispensing.

We modeled satisfaction with the mail-order model (ie, very satisfied compared with all others) using mixed-effects multivariable logistic regression; variables included participant age, race and ethnicity, education, parity, prior abortion experience, gestational duration, satisfaction with package delivery time, package condition, and whether confidentiality was maintained during shipping. We included race and ethnicity, with White as the reference category because we hypothesized that experiences with racism in health care settings, including in the context of the current abortion, may affect satisfaction, and because individuals of other race and/or ethnicity have been shown to be more likely to experience race-based discrimination in health care.^24^ We adjusted for clustering by clinic site as a random effect in mixed-effects regression models.

The target sample size was based on the primary outcome measures of effectiveness and acceptability, assuming 10% loss to follow-up, 10% adjustment for clustering, a 5% noninferiority margin, and a 2-sided α = .05. The planned sample size was a minimum of 440 participants, which would provide a final analytic sample of approximately 400 patients and 98%, 79%, and 77% power to assess the proportion with a complete abortion, the proportion who would use the mail-order service again, and the proportion who reported being satisfied or very satisfied with the medication abortion, respectively. Data were analyzed from August 2022 to December 2023 using Stata, release 17.0 (StataCorp LLC).

## Results

We obtained clinical outcome data from clinic records and self-reported survey data for 510 abortions among 506 participants (median [IQR] age, 27 [23-31] years; 506 [100%] female; 194 [38.3%] Black, 88 [17.4%] Hispanic, 141 [27.9%] White, and 45 [8.9%] multiracial/other individuals). Additional self-reported participant characteristics are presented in Table 1.

**Table 1.** Sociodemographic Characteristics of Study Participants Having Medication Abortion and Receiving Mifepristone From a Mail-Order Pharmacy, 2020 to 2022^a^

Characteristic	No. (%)
Total participants	506
Age group, y	
15-19	30 (5.9)
20-24	144 (28.5)
25-29	161 (31.8)
≥30	171 (33.8)
Age, median (IQR), y	27 (23-31)
Race and ethnicity	
Black	194 (38.3)
Hispanic	88 (17.4)
White	141 (27.9)
Multiracial/other race^b^	45 (8.9)
Missing data	38 (7.5)
Educational level	
High school or less	170 (33.6)
Some college or professional school	206 (40.7)
College or advanced degree	112 (22.1)
Missing data	18 (3.6)
Parity	
Nulliparous	189 (37.4)
Parous	317 (62.6)
Prior abortion experience	
None	249 (49.2)
Medication abortion	149 (29.4)
Procedural abortion only	93 (18.4)
Missing data	15 (3.0)

^a^ Among participants who took medications and had clinical outcome data available (unit of analysis is the number of individuals in the study).

^b^ Other race included Cape Verdean; Native Hawaiian or Pacific Islander; American Indian or Alaska Native; and those who did not report this information.

The median (range) number of participants per study site was 18 (2-209) overall, with 48 (26-209) among abortion sites and 7 (2-18) among primary care sites. Of 510 abortions, we obtained completed day-3 survey data for 495 participants (97%) and day-14 survey data for 477 (94%).

All participants received their medications by mail and 436 (85.5%; 95% CI, 82.2% to 88.4%) received the package within 3 days of enrollment (Table 2). The pharmacy sent a second package to 5 participants who experienced delivery delays. Delivery time was reported to be reasonable by 467 recipients (94.3%), whereas 27 (5.5%) reported it was too long (Table 3). The package was reported to be in good condition by 482 participants (97.4%) and damaged by 12 (2.4%); none reported damage to the medications. In addition, 486 respondents (98.2%) reported that their confidentiality was maintained during the shipping and delivery process; 8 reported that their confidentiality was compromised when another person saw the package (n = 5), opened the package (n = 2), or observed their pregnancy symptoms or medication adverse effects (n = 3).

**Table 2.** Clinical Outcomes and Regimen for Participants Having Medication Abortion and Receiving Mifepristone From a Mail-Order Pharmacy, 2020 to 2022 (N = 510)^a^

Clinical outcome variables	No. (%)
Recruitment site	
Primary care clinic	47 (9.2)
Abortion clinic	463 (90.8)
Gestational duration at initial clinic visit, d^b^	
≤49	360 (70.6)
50-56	93 (18.2)
57-63	57 (11.2)
Medication delivery time (from prescription to package delivery), d	
≤3	436 (85.5)
4-7	71 (13.9)
>7	3 (0.6)
Gestational duration at date of taking mifepristone, d	
≤49	260 (51.0)
50-56	136 (26.7)
57-63	72 (14.1)
64-70	29 (5.7)
>70	3 (0.6)
Missing data	10 (2.0)
Gestational duration at mifepristone, median (IQR), d	49 (44-55)
Route of misoprostol administration	
Buccal	486 (95.3)
Vaginal	5 (1.0)
Missing data	19 (3.7)
Interval between mifepristone and misoprostol, h	
<24	5 (1.0)
24-48	491 (96.3)
Missing data	14 (2.7)
Initial dose of misoprostol, 800 µg	483 (94.7)
Missing data	27 (5.3)
Abortion outcome	
Complete with medications only	499 (97.8)
With repeat dose of misoprostol^c^	27 (5.3)
Unsuccessful medication abortion^d^	11 (2.2)
Incomplete abortion	5 (1.0)
Ongoing pregnancy	6 (1.2)
Confirmation of abortion completion, total No.	499
Followed-up with study clinic, No.	336
Ultrasonography	121 (36.0)
Serial serum human chorionic gonadotropin testing	14 (4.2)
Negative urine pregnancy test (in clinic)	9 (2.7)
Clinical history and home urine pregnancy test (telephone visit)	141 (42.0)
Clinical history alone (telephone visit)	51 (15.2)
No follow-up with clinic but indicated completion on survey, No.	163
Negative result of home urine pregnancy test	117 (71.8)
Reported that clinic said abortion was complete	4 (2.5)
Reported an ultrasonography	2 (1.2)
Reported completion based on history alone^e^	40 (24.5)
Normal menstrual period returned	4 (2.5)
No more pregnancy symptoms	33 (20.2)
Other, eg, heavy bleeding/passed tissue	2 (1.2)
Self-reported but no reason given	6 (3.7)

^a^ Among participants who took medications and had clinical outcome data available. Includes 4 participants who had 2 abortions in study (unit of analysis is the number of abortions).

^b^ Ultrasonography was used to assess gestational duration in 487 participants; 23 had only clinical assessment with physical examination.

^c^ Nine participants were prescribed a second dose initially, and 18 were prescribed a second dose at follow-up for incomplete abortion.

^d^ All had a procedural abortion except 1 participant who chose to continue the pregnancy.

^e^ Some participants reported more than 1 option.

**Table 3.** Acceptability and Satisfaction at Day 3 Survey Among Study Participants Having Medication Abortion and Receiving Mifepristone From a Mail-Order Pharmacy, 2020 to 2022 (n = 495)^a^

Acceptability variables	No. (%)
Satisfaction with receiving medications by mail	
Very satisfied	452 (91.3)
Somewhat satisfied	26 (5.3)
Neither satisfied nor dissatisfied	7 (1.4)
Somewhat dissatisfied	4 (0.8)
Very dissatisfied	6 (1.2)
Acceptability of medication delivery time	
Reasonable	467 (94.3)
Too long	27 (5.5)
Missing data	1 (0.2)
Condition of package	
Good condition (no evidence of tampering)	482 (97.4)
Damaged (eg, opened, punctured, crushed)	12 (2.4)
Missing data	1 (0.2)
Location where package was received	
Home address	448 (90.5)
Family, friend, or partner’s house	18 (3.6)
Study clinic	4 (0.8)
Work address	2 (0.4)
Somewhere else (not specified)	16 (3.2)
Missing data	7 (1.4)
Confidentiality maintained during shipping	
Yes	486 (98.2)
No, confidentiality was compromised	8 (1.6)
Missing data	1 (0.2)
Adequate information received from clinic	
Yes	494 (99.8)
No, I would have liked more information	1 (0.2)
Current vs previous medication abortion experience (among those who had previous medication abortion), No.	150^b^
This time was better	82 (54.7)
Same	54 (36.0)
Last time was better	4 (2.7)
Not sure	6 (4.0)
Missing data	4 (2.7)

^a^ Among participants who had clinical outcome data available and completed the day 3 survey (unit of analysis is the number of abortions).

^b^ One participant who enrolled twice reported prior medication abortion experience.

Complete medication abortion occurred for 499 of 510 cases (97.8%; 95% CI, 96.2% to 98.9%), including for 27 participants (5%) who took an additional dose of misoprostol. This compares favorably with a complete abortion rate of 97.4% cited in the mifepristone label^18^ (risk difference, 0.004; 95% CI, −0.009 to 0.017; *P* = .58). eTable 1 in Supplement 1 shows effectiveness by gestational duration. Eleven participants (2.2%) had an unsuccessful medication abortion; 10 obtained a vacuum aspiration for incomplete abortion (n = 5) or for ongoing pregnancy (n = 5). One participant chose to continue the pregnancy after taking the medications and subsequently reported the uncomplicated delivery of a healthy newborn.

There were 24 AEs (4.7%; 95% CI, 3.0%-7.0%) for which patients sought care for symptoms that were possibly, probably, or definitely related to the medication abortion. These included unscheduled visits for symptoms such as bleeding, pain, nausea, vomiting, infection, and diarrhea. Seventeen AEs included an emergency department visit; in 10 of these visits, the patient received treatment such as analgesics, antibiotics, intravenous fluids, or vacuum aspiration for incomplete abortion. Three serious AEs occurred (0.6%; 95% CI, 0.1%-1.7%), all of which involved hospitalization: 1 participant received a blood transfusion for hemorrhage with incomplete abortion, 1 received antibiotics for an infection with incomplete abortion, and 1 received no treatment. There were no AEs related to mail-order pharmacy dispensing.

Nearly all participants (478 of 495 [96.6%; 95% CI, 94.6 to 98.0]) reported they were very satisfied (91.3%) or somewhat satisfied (5.3%) with mail-order dispensing (Table 3). Of 477 participants, 431 (90.4%; 95% CI, 87.3% to 92.9%) reported that they would use it again for a future medication abortion if needed; this proportion is similar to a published estimate^23^ indicating that 89.7% of individuals accessing the service with in-person dispensing said they would use medication abortion again (risk difference, 0.007; 95% CI, −0.02 to 0.04; *P* = .67).

Participants who were less than very satisfied with mail-order dispensing (n = 43; Table 3) provided open-ended responses describing what could be improved. Approximately half said the timing of delivery could be more aligned with expectations set by the clinic. Some suggested providing patients with package-tracking information. Three participants said they would have preferred more privacy in the delivery process (eg, better package placement, hand-to-hand delivery, and avoiding the word “abortion” on documents included in the package).

Regarding the medication abortion experience overall, 91.2% (435 of 477; 95% CI, 88.3%-93.6%) said they were very satisfied (80%) or satisfied (11%) (Table 4), which is similar to a published estimate^22^ in which satisfaction of medication abortion with in-person dispensing was 88.4% (risk difference, 0.028; 95% CI, 0.001-0.055; *P* = .06). In multivariable regression analysis, those who reported the delivery time was too long (adjusted odds ratio, 0.04; 95% CI, 0.01-0.10) or that confidentiality was compromised (adjusted odds ratio, 0.05; 95% CI, 0.01-0.32) had significantly lower odds of reporting satisfaction with mail-order dispensing (eTable 2 in Supplement 1).

**Table 4.** Acceptability and Satisfaction at Day 14 Survey Among Participants Having Medication Abortion and Receiving Mifepristone From a Mail-Order Pharmacy, 2020 to 2022 (n = 477)^a^

Acceptability variables	No. (%)
Overall satisfaction with medication abortion	
Very satisfied	382 (80.1)
Somewhat satisfied	53 (11.1)
Neither satisfied nor dissatisfied	31 (6.5)
Somewhat dissatisfied	6 (1.3)
Very dissatisfied	5 (1.0)
Would recommend medication abortion to a friend	
Yes	431 (90.4)
No	14 (2.9)
Not sure	32 (6.7)
Would recommend mail-order dispensing to a friend	
Yes	447 (93.7)
No	5 (1.0)
Not sure	23 (4.8)
Missing data	2 (0.4)
Would prefer to receive medications by mail for future medication abortion	
Yes	431 (90.4)
No	20 (4.2)
Not sure	26 (5.5)

^a^ Among participants who had clinical outcome data available and completed the day 14 survey (unit of analysis is the number of abortions).

## Discussion

We found that medication abortion with mail-order pharmacy dispensing of medications after an in-person assessment for eligibility was effective and acceptable to patients with comparable findings to other studies of medication abortion with in-person dispensing.^18,22,23^ The mail-order model was feasible, with 85.5% of participants receiving the medications within 3 days and 99.4% within 7 days. Although the study was not powered to estimate safety outcomes, we observed a low prevalence of serious AEs (0.6%, 95% CI 0.1%-1.7%). This is similar to a report of 11 319 medication abortions with in-person dispensing in California in 2009 to 2010 that found only 0.3% had a major complication.^25^

This study adds to a growing body of literature demonstrating that medication abortion can be provided safely and effectively using models of care that do not involve a clinician dispensing mifepristone in person. Another US study found that medication abortion with mifepristone dispensed from a brick-and-mortar pharmacy was effective and acceptable to patients with a low prevalence of AEs.^26^ Other US studies of medication abortion with telehealth evaluation for eligibility and medications mailed either by the clinician or using a mail-order pharmacy similarly have found these models to be effective (estimates of complete abortion range from 93% to 99%), acceptable to patients (satisfaction ranges from 96% to 100%), and safe (serious AEs range from 0% to 1%).^6,27,28,29,30,31,32^ This body of research supports the FDA decision in 2021 to permanently remove the in-person dispensing requirement for mifepristone.

Although overall satisfaction with mail-order dispensing was high, participants noted areas for improvement, primarily related to meeting expectations regarding the timing and tracking of medication delivery. Mechanisms for ensuring speedy and confidential delivery are already used by major shipping companies, including for the delivery of pharmaceuticals, and should be no different for abortion medications. A recent analysis of a retrospective cohort study^33^ found that mailing pills to patients was not significantly associated with delays in obtaining medication abortion compared with receiving pills in the clinic.

With the severe restrictions on abortion care imposed since the *Dobbs* decision,^3^ pharmacy dispensing of mifepristone has an important role to play in improving access. For both patients in states where abortion remains legal and those in states with restrictions who must travel for services, expanded access to medication abortion may reduce delays to care and congestion at abortion clinics where procedural abortion is provided.^34^ Pharmacy dispensing also could enable more practitioners, including primary care clinicians, to provide medication abortion by removing the requirement to stock mifepristone. Offering this service in the primary care setting, where communities that historically experience barriers to care can most easily access reproductive health services, could help to normalize medication abortion and improve continuity of care, as well as expand access.^35^ So far, only a small number of brick-and-mortar pharmacies have become certified to dispense mifepristone.^36^ Mail-order pharmacy dispensing could offer convenience and timely access to abortion medicines for patients who live far from certified brick-and-mortar pharmacies.

When combined with telehealth services for eligibility screening and counseling, mail-order dispensing of mifepristone allows for fully remote medication abortion. Although not the focus of the present study, fully remote medication abortion could likely expand access to a greater extent than models requiring in-person eligibility assessment. A recent analysis^37^ found that telehealth provision of medication abortion helped certain patients obtain a timely abortion, including younger patients, those experiencing food insecurity, those living in rural areas, or those who averted traveling more than 100 miles to the nearest abortion clinic.

### Strengths and Limitations

This study has several strengths, including recruiting from both established abortion clinics and primary care clinics new to abortion care in a range of geographic settings, as well as the low loss to follow-up (3.8%). The study also has limitations. The intervention was not randomized, which may limit generalizability. Clinical sites that agreed to participate had at least 1 motivated clinician and a supportive administrative environment and were typically located in states with relatively few abortion restrictions. Patients who agreed to participate in the study were open to mail-order dispensing, and this option may not be acceptable or feasible for all patients. The study also was not powered to precisely estimate safety outcomes. In addition, satisfaction with services may be overestimated due to social desirability bias.

## Conclusions

The findings of this cohort study on the effectiveness and acceptability of medication abortion using a mail-order pharmacy to dispense mifepristone were comparable with published studies of in-person dispensing. This study adds to the substantial body of evidence supporting the FDA decision to remove the in-person dispensing requirement for mifepristone. Building on this policy change, efforts are needed to expand pharmacy dispensing of mifepristone and telehealth provision of medication abortion, and to test other innovative strategies to reduce barriers to this critical element of comprehensive health care.

## Notes

Supplement 1. eTable 1. Abortion outcome by gestational duration among people with clinical outcome data, 2020-2022 eTable 2. Factors associated with being “very satisfied” with the mail-order model among people with clinical outcome data, 2020-2022 (n=489)

Supplement 2. Data Sharing Statement
