# Supplementary material for: Mail-Order Pharmacy Dispensing of Mifepristone for Medication Abortion After In-Person Screening
Source: JAMA Intern Med. 2024 May 13;184(8):873–81. doi: 10.1001/jamainternmed.2024.1476 (PMC11091818; doi:10.1001/jamainternmed.2024.1476)
Supplement: Supplement 1. — eTable 1. Abortion outcome by gestational duration among people with clinical outcome data, 2020-2022 eTable 2. Factors associated with being “very satisfied” with the mail-order model among people with clinical outcome data, 2020-2022 (n=489) [file jamainternmed-e241476-s001.pdf]

Supplemental Online Content

Grossman D, Raifman S, Morris N, et al. Mail-Order Pharmacy Dispensing of Mifepristone for Medication Abortion After In-Person Screening. *JAMA Intern Med.* Published online May 13, 2024. doi:10.1001/jamainternmed.2024.1476

eTable 1. Abortion outcome by gestational duration among people with clinical outcome data, 2020-2022

eTable 2. Factors associated with being "very satisfied" with the mail-order model among people with clinical outcome data, 2020-2022 (n=489)

This supplemental material has been provided by the authors to give readers additional information about their work.

© 2024 Grossman D et al. *JAMA Internal Med.*

eTable 1. Abortion outcome by gestational duration among people with clinical outcome data, 2020-2022

Gestational duration at date of taking mifepristone (days)	Abortion outcome					
	Complete with medications only (n = 499)		Incomplete abortion, had vacuum aspiration (n = 5)		Ongoing pregnancy (n = 6)[a]	
≤49	259	99.6%	1	0.4%	0	0%
50-56	132	97.1%	2	1.5%	2	1.5%
57-63	68	94.4%	2	2.8%	2	2.8%
64-70	28	96.6%	0	0%	1	3.4%
>70[b]	3	100%	0	0%	0	0%
Unknown	9	90.0%	0	0%	1	10.0%

[a]1 participant opted to continue the pregnancy.

[b]2 participants had a gestational duration of 71 days, and 1 participant had a gestational duration of 72 days

© 2024 Grossman D et al. *JAMA Internal Med.*

eTable 2. Factors associated with being "very satisfied" with the mail-order model among people with clinical outcome data, 2020-2022 (n=489)

**Independent variables**	**aOR (95% CI)**
**Age (years)**	
15-19	1.4 (0.24, 8.00)
20-24	(ref)
25-29	1.78 (0.67, 4.77)
≥30	2.25 (0.74, 6.86)
**Race and ethnicity**	
Non-Hispanic Black	0.52 (0.19, 1.42)
Hispanic	1.06 (0.27, 4.17)
Non-Hispanic White	(ref)
More than one race & "other" race	0.42 (0.11, 1.57)
Unknown race / ethnicity	0.38 (0.08, 1.95)
**Education**	
High school or less	(ref)
Some college or professional school	0.43 (0.17, 1.12)
College or advanced degree	0.67 (0.20, 2.26)
**Parity**	
Nulliparous	(ref)
Parous	1.35 (0.53, 3.45)
**Prior abortion experience**	
None	(ref)
Medication abortion	0.95 (0.38, 2.34)
Procedural abortion only	1.61 (0.47, 5.44)
**Gestational duration at initial clinic visit (days)**	
≤49	(ref)
50-56	0.95 (0.34, 2.63)
57-63	0.89 (0.29, 2.72)
**Acceptability of medication delivery time**	
Reasonable	(ref)
Too long	**0.04 (0.01, 0.10)**
**Condition of package**	
Good condition (no evidence of tampering)	(ref)
Damaged (opened, punctured, crushed, etc.)	0.95 (0.09, 9.48)
**Confidentiality maintained during shipping**	
Yes	(ref)
No, confidentiality was compromised	**0.05 (0.01, 0.32)**

© 2024 Grossman D et al. *JAMA Internal Med.*

Filename: IOI240027supp1_edited.docx
Directory: C:\Program Files (x86)\eXtyles
Template: C:\Users\sthollan\AppData\Roaming\Microsoft\Templates\Normal.dotm
Title:
Subject:
Author: Grossman, Daniel
Keywords:
Comments:
Creation Date: 4/3/2024 4:15:00 PM
Change Number: 2
Last Saved On: 4/3/2024 4:15:00 PM
Last Saved By: SR Holland
Total Editing Time: 0 Minutes
Last Printed On: 4/3/2024 4:16:00 PM
As of Last Complete Printing
Number of Pages: 3
Number of Words: 375 (approx.)
Number of Characters: 2,143 (approx.)
