# Supplementary material for: Mail-Order Pharmacy Dispensing of Mifepristone for Medication Abortion After In-Person Screening
Source: JAMA Intern Med. 2024 May 13;184(8):873–81. doi: 10.1001/jamainternmed.2024.1476 (PMC11091818; doi:10.1001/jamainternmed.2024.1476)
Supplement: Supplement 2. — Data Sharing Statement [file jamainternmed-e241476-s002.pdf]

Data Sharing Statement

Grossman. Mail-Order Pharmacy Dispensing of Mifepristone for Medication Abortion After In-Person Screening. *JAMA Intern Med*. Published May 13, 2024.
doi:10.1001/jamainternmed.2024.1476

Data
Data available: No

Additional Information
Explanation for why data not available: Given the very sensitive nature of the data collected, data will not be shared.
